# Proceedings of the Nineteenth Annual Meeting of the Northern Ohio Dental Association

**Published:** 1878-10

**Authors:** 


### PROCEEDINGS OF THE NINETEENTH ANNUAL MEETING OF THE NORTHERN OHIO DENTAL ASSOCIATION.

*Continued from page 347.*

The second subject, Necrosis and Exfoliation of the Alveolar Processes, Causes and Treatment, was taken up for consideration.

A case under the care of Dr. Miller, of Elyria, had been examined during the day by some of the gentlemen present, and on request he made the following statement of the case to the Association:

The case is that of a lady about forty years of age, healthy and vigorous. Abscess in alveolar substance in upper right maxilla, which has given her trouble at various times during the past eight years. It had been opened by several dentists and once by a physician and treated each time; the right lateral incisor has been removed for a year; the right central is devitalized; the opening is over the location where the lateral incisor had been; a probe can be made to touch the right central and right cuspid, and to extend upward to the wall of the nares, the alveolar process being lost by the action of caries; at the time of examination there was a discharge from the opening of a serous character, and a small quantity of unhealthy pus; learned nothing from the patient that would indicate the cause.

Dr. Buffett of Cleveland, said: It is hard to say from the statement that it is a case of necrosis. Necrosis is the death, of a part of bone from the lack of nutrition and blood. Nature in her effort to heal, w'll absorb some of the living tissue adjoining the necrossed part, but the dead bone is never absorbed. Caries of the bone, on the other hand is molecular death, the particles dying one after another. It never cures itself. Necrosis does. It is difficult to say which this case is. If it is caries, it is modified by the treatment it has received. Necrosis and caries sometimes exist together, and both may have existed in this case. It was undoubtedly caused by inflammation. Caries is produced by a low vitality, while high vitality produces necrosis. We have serous tumors as a result of roots left in the cavity. The present case is of that nature. It is an open serous cyst. The treatment is to break up the membrane which secretes the pus, by bringing in contact with it something to cauterize the surface. There may be a hereditary taint of scrofula. A robust face is not a sure indication of sound health. She should be put under tonic treatment, as iron, meats, etc., taking away starchy foods.

For cauterizing, almost any acid is good, and may be diluted. I should recommend, and above all other applications, the chloride of zinc. The old treatment was with nitrate of silver, but it always leaves a deposit of silver which interferes with the formation of new membrane. Other acids may destroy the periosteum, but new membrane will be produced. If these remedies fail, open thoroughly with a knife. Where a root is left, and serous formation produced, it can not be cured until opened up and the pus found.

Dr. Horton, of Cleveland, said this case might be the result of years of imperfect health; it might result from fever, or from smallpox, etc., which he had frequently known to produce necrosis. There is frequently a withdrawal of nutrition from certain parts arising from high, living, and lack of exercise, the system having in it more nutriment than it can take care of, and nature being over taxed finally refuses to supply the needs of all parts of the system. Either of these causes might produce the result under consideration. There seemed to be no syphilitic taint, but it rather arose from withdrawal of nutrition. If the necrossed part were removed by the use of forceps the evil would cure itself.

These remarks upon the case presented by Dr. Miller, were interspersed by numerous illustrations of cases that had come under the observation of the gentlemen present, and considerable informal discussion followed, but of such a nature that it can not be easily embodied in this report.

EVENING SESSION.

The Association met again in the rooms of Dr. Kelsey at half-past seven o'clock.

The report of the Examining Committee was received, recommending the admission to membership of Messrs Brown, of Cleveland, Chandler, of Sandusky, and Craiger, of Fremont.

The gentlemen named were admitted by vote of the Association.

The Treasurer's report was read, and on motion adopted and placed on file.

After the reports of the committees had been received, the discussion of the subject taken up in the afternoon was continued.

Dr. Robinson, of Cleveland, said the subject was capable of large discussion and treatment. These diseases give as much trouble as any other with which dentists have to deal. There are frequently cases of necrosis presented where there seems to be no cause for abscess, yet it is there. I sometimes think the necrosis must be produced by a sharp blow upon the jaw. I never opened a necrosed tooth without finding the nerve dead. Nervous diseases may produce necrosis, but I do not believe it. My treatment is to open the tooth and cleanse it thoroughly.

Dr. Buffet—We often meet with necrosis and caries without being able to determine the cause. We find in children often, abscesses in the alveolar process opening to the roots of the teeth. They are caused by inflammation arising from scrofulous or syphilitic taint. If the trouble only afflicts the outer plate it is better not to extract the tooth for a year or so. After a tooth is denuded of the periosteum, we can not expect much bone to be made. Whenever the opening is in the external plate, it may be easily remedied. The attachment which the temporary tooth has with the periosteum may interfere with the growth of the permanent tooth, and where we can it is better to protect it, and especially with the incisors; the molars may be extracted with far less harm. In children the initiatory substance is a decaying pulp—a poison peculiar to the disease—which acts on the alveolar process. The treatment is to fill the roots with a soft material and treat the part with gentle stimulents. We are apt to over-treat. We should only assist nature. Acids stimulate nature to do her work, but we must not apply them too freely. Some of the most successful practitioners have only used warm water, or alcohol. The only object is to thoroughly cleanse the tooth. Cleansing is the main thing.

We seldom find necrosis in older persons together with ulceration from the tooth. It is more frequently produced by blows and stimulating medicines, which deprive that portion of its nutriment and death results. Chloride of zinc, though good, may, and often does, produce this result. In using acids we must be careful and judge by the character of the patient as to the strength and quantity to be used. Arsenic has a persistent result, but it does not exfoliate readily. Some have said that arsenic does not belong to the dentist. That is for you to say. Its careless use produces necrosis, and never cures. Blows, fractures, etc., produce necrosis, and it may be manifest only in years from the time of the injury. If necrosis follows as the result of a very slight blow, you may be sure there is a hereditary taint lurking in the system. A mere wedging of the tooth for the purpose of filling it has produced it. In treating such cases we must trust to time and soothing applications; but we must closely watch the patient and judge what treatment may be judicious.

These remarks were supplemented by a running discussion by Drs. Horton, Brown, Craiger, Miller, Pool and others after which the meeting was adjourned.

### WEDNESDAY—MORNING SESSION.

The meeting was called to order, at nine-thirty, and the officers for the ensuing year elected, as follows:

President, J. W. Lyder, of Akron; Vice-president, J. F. Siddell, of Oberlin; Corresponding Secretary, L. Buffett, Cleveland; Treasurer, J. E. Robinson, Cleveland; Recording Secretary, L. C. Kelsey, Elyria.

The President-elect appointed the following committee to ascertain the quota of delegates to the national convention, viz.: Drs. Chandler, Robinson and Pool. The committee retired and on their reappearance named the following gentlemen as delegates: J. W. Lyder, E. W. Pool, J. C. Merritt, Ira Brown, D. P. Jennings, L. Buffett and Fred. Craiger.

The following named gentlemen were appointed as Executive Committee: J. E. Robinson and D. P. Jennings.

The Board of Examiners was filled as follows: Messrs. C. Buffett, F. Whistler and Ira Brown.

Previous to the opening hour an interesting case, under the care of Dr. Kelsey, had been examined and operated upon in the presence of the Association.

On motion of Dr. Robinson, the third subject laid down for discussion was passed, and the case examined in the morning taken up for consideration. We give a statement of the case as made by Dr. Cushing, a resident physician of Elyria. A gentleman aged sixty-five; tooth broken off twenty years ago; root extracted six or seven years ago, there being no toothache or neuralgia previous; there was, however, an alveolur abscess on the outer plate, opposite the point lanced, five or six years before the root was extracted. Soon after extraction of the root he began to feel a slight pain at times in the vicinity of the mental foramen, but not till four years ago did it become severe; since then the longest interval of entire freedom from pain was two weeks during the summer of 1877. He has no pain at all during the night. Washing the face in the morning almost always brings on a paroxysm of pain; it is less likely to at other times of day. Eating in the morning almost always produces a paroxysm, and often eating at other times brings about the same result. He is never capable of masticating fearlessly. Some days the pain is continuous and so severe that it is difficult to read aloud, to converse or to expectorate. During the early years the pain was noticed over the ramus of the jaw and up to the meatus. Three or four times last fall the pain extended from the inner canthus of the eye downward to the *alæ nasi*, but none before or since. The tooth extracted was the left inferior second bicuspid.

This case was of much interest to the Association from its long standing and almost constant pain. By dissecting away the gum and using the forceps some loose pieces of the alveoler process were removed; and it was then discovered by Dr. Robinson that the adjoining molar, free from decay, was devitalized, and it was the opinion of most of the members that this was the cause of the trouble, and its extraction was recommended.

### AFTERNOON SESSION.

The Association met at two o'clock. On motion a clinic was held in the afternoon under charge of Dr. Robinson. The subject was filling teeth.

On motion the fourth subject of discussion was taken up and considered for about an hour. There was very little disagreement and the substance of what was said was that each and every dentist must exereise his own judgment as to the materials to be used in filling teeth; that such material should be used as will produce the best results; all circumstances being equal gold is the best material that can be used; that for filling children's teeth tin is preferable, being less subject to thermal changes.

On motion a vote of thanks was tendered Dr. Kilsey for the use of his rooms for the meetings of the Association.

On motion the Association adjourned to meet in Cleveland on the second Tuesday in May, 1879.

